# A survey of knowledge and perceptions of ADHD and autism spectrum disorder in the workplace at a large corporation

**DOI:** 10.1038/s41598-025-17470-8

**Published:** 2025-10-02

**Authors:** Javier Quintero, Alberto Rodríguez-Quiroga, Jesús Medina, Marta Galvez-Fernandez, Javier Nuevo, Carla Ruiz, Elena Sánchez, Victoria Alonso, Ana Pérez Domínguez

**Affiliations:** 1 Hospital Universitario Infanta Leonor, Madrid, Spain; 2 Departamento de Medicina Legal, Psiquiatría y Patología, Universidad Complutense de Madrid, Madrid, Spain; 3 Biopharmaceuticals Medical, AstraZeneca , Madrid, Spain; 4 Human Resources, AstraZeneca , Madrid, Spain; 5 Unidad de Neurodesarrollo, PsiKids , Madrid, Spain

**Keywords:** Neurodiversity, Attention-deficit/hyperactivity disorder (ADHD), Autism spectrum disorder (ASD), Workplace inclusion, Innovation, Diversity, Survey, Social neuroscience, ADHD, Autism spectrum disorders

## Abstract

Neurodevelopmental conditions, such as attention-deficit/hyperactivity disorder (ADHD) and autism spectrum disorder (ASD), affect millions globally and are often misunderstood in professional environments. Embracing neurodiversity is essential for inclusivity and leveraging the unique strengths of these individuals. This study evaluated employees’ knowledge and perceptions of neurodiversity, focusing on ADHD and autism in large corporations, to identify knowledge gaps and propose strategies to create more inclusive workplaces. Cross-sectional survey of 880 employees from AstraZeneca and Alexion in Spain was conducted in July 2024. It assessed knowledge of ADHD and autism, perceptions of working with neurodivergent individuals, and preferences for educational interventions. Data were collected via self-administered questionnaire and analyzed descriptively and analyzed using descriptive statistics to summarize frequencies, proportions, and central tendency measures. Awareness of ADHD (98.9%) and autism (98.1%) was high, but misconceptions persisted, with ~ 20% misidentifying intellectual disability as a symptom of autism, and restricted interests as a sign of ADHD. Comfort with potentially working with neurodivergent colleagues was rated 7.4/10, yet 60.6% felt that workplaces were inadequately adapted. Preferred educational approaches included school talks (87.5%) and social media (67.6%). Despite positive attitudes, knowledge gaps regarding neurodevelopmental disorders persist. Targeted interventions are essential to emphasize the strengths of neurodivergent individuals and foster adaptable, supportive workplaces that promote inclusivity and innovation.

**Supplementary Information:**

The online version contains supplementary material available at 10.1038/s41598-025-17470-8.

## Introduction

As we progress toward a mindset where diverse talent is increasingly valued, the workplace emerges as a pivotal arena for embracing inclusive and transformative integration. Among the most powerful yet often underestimated dimensions of this shift are the inclusion of neurodivergent individuals, such as those with conditions like attention-deficit/hyperactivity disorder (ADHD) and autism spectrum disorder (ASD) (Based on the literature, we will use the term “autism” in most instances throughout the manuscript autistic community members and scholars find the term ASD to be negative and prefer the term “autism.” (Bottema-Beutel, K., Kapp, S. K., Lester, J. N., Sasson, N. J., & Hand, B. N. (2021). Avoiding ableist language: Suggestions for autism researchers. Autism in adulthood, 3(1), 18–29), into professional environments^1^. These conditions, categorized as neurodevelopmental disorders, are characterized by atypical brain development that affects cognitive, behavioral, and social functioning, typically manifesting during the developmental period. This natural diversity in human brain functioning is referred to as neurodiversity. As a result, neurodivergent individuals often present distinctive cognitive, behavioral, and social patterns that stand out in conventional work settings^1^. They affect millions of people worldwide, with estimates suggesting that 5%−7% of adults have ADHD and 1%−2% are on the autism spectrum^2^. In Europe, the prevalence of such disorders is similar^3^.

Despite a growing awareness of ADHD and autism in our society, pervasive misconceptions continue to hinder the acceptance of neurodivergent individuals in the workplace. Both conditions are often associated with challenges in executive functioning, such as planning, organization, and self-regulation difficulties^4^, which can be misinterpreted as barriers to professional success. While these difficulties may impact task completion, this misinterpretation arises when such challenges are viewed solely as deficits rather than as a mismatch between an individual’s cognitive profile and traditional workplace demands. With appropriate support and accommodations, many neurodivergent individuals successfully leverage their strengths to perform effectively^5–7^.

ADHD is often associated with difficulties in time management, attention regulation, and impulsivity, while individuals with autism may experience challenges in social communication and sensitivity to external stimuli^8–10^. Yet this limited perspective overlooks the unique strengths that these individuals may bring to the table. People with neurodivergent traits often excel in areas such as pattern recognition, attention to detail, and complex problem-solving^11,12^, making them valuable assets in innovative environments. Recent research has highlighted not only these strengths but also that awareness and active use of these strengths are linked to better wellbeing and quality of life in individuals with autism and ADHD^5–7^.

Thus, embracing neurodiversity is not merely a moral imperative but a strategic advantage for companies aiming to foster creativity, resilience, and forward-thinking cultures. As companies adopt a more inclusive approach, they unlock not only the potential of their neurodivergent employees but also the innovative capacity of their entire workforce^13,14^. In this context, neurodiversity should be seen not as a matter of social responsibility alone but as an opportunity for growth and innovation that challenges outdated stigmas and aligns with progressive business values.

Some regions, such as the United States and the United Kingdom, have begun to integrate workplace policies that support neurodivergent employees^15,16^. However, significant work remains to be done in countries like Spain, where workplace policies to support neurodivergent individuals are still in development. While, academic and educational initiatives have made progress in integrating neurodivergent individuals into specific sectors^17^, the professional sphere still needs to be explored. In order to build a strong foundation for inclusion, we must promote environments that reflect scientific understanding and are aligned with the real needs of organizations and neurodivergent individuals.

This paper aims to open pathways toward a deeper understanding of neurodiversity in professional settings by exploring the perceptions and experiences of neurodiversity in the workplace and the opportunities for appropriate employment policies. This research, conducted in two leading organizations, presents key findings from an in-depth survey of employees on the current challenges and the benefits of embracing neurodiversity in the workplace. Ultimately, it contributes to the broader conversation on how businesses can create environments where neurodiverse talent is valued as a necessity for innovation, growth, and inclusion in the modern world.

## Methods

### Study design

This study was based on a cross-sectional survey of AstraZeneca and Alexion (a subsidiary company of AstraZeneca) employees in Spain, conducted in July 2024. The primary objective was to assess employees’ knowledge of ADHD and autism and their perceptions of working with neurodivergent individuals, in order to raise awareness of neurodiversity and underscore the importance of creating suitable workspaces. Participants were recruited from several corporate locations, including AstraZeneca Spain, the AstraZeneca Global Hub in Barcelona, and Alexion Spain/Global, ensuring diversity within the sample. No formal inclusion or exclusion criteria were applied, as the survey was distributed anonymously to all employees of the company. Participation was entirely voluntary, and no data were collected that could identify individual respondents.

### Survey instrument

Data were collected using a self-administered, pre-designed questionnaire hosted on Microsoft Forms. The twenty-six questions and response options were mainly developed by JQ and ARQ, experts in ADHD and autism and based on previous studies that have shown variability in knowledge and perceptions regarding ADHD and autism across different international contexts^18–21^. Specifically, the questionnaire was constructed based on prior international surveys and the authors’ clinical and research expertise. While formal psychometric validation was not performed, all items were reviewed by two independent experts in ADHD and autism for content relevance and clarity.

Questionnaire was available in both English and Spanish to facilitate broader participation. Participants in this study were required to be employees of AstraZeneca or Alexion. The questionnaire was sent to 1168 individuals and comprised three core areas:

1. Demographic information: This section collected participants’ age, gender, education level, and professional background.
2. Knowledge of ADHD and ASD: Participants answered a series of questions designed to test their understanding of the symptoms of ADHD and autism. Example items include: “Which of the following are typical features of ADHD?” (multiple choice) and “Which symptoms are commonly associated with autism?” (multiple choice).
3. Perceptions of neurodivergent individuals: Participants provided their views on working with individuals who have ADHD or autism, including comfort levels and perceived challenges. Example items assessing perceptions include: “How comfortable would you feel working with a person with ADHD?” (Likert scale from 0 to 10), and “What adaptations do you think would improve inclusion for neurodivergent colleagues?” (open-ended).

The questionnaire included yes/no questions, multiple-choice questions, open-ended questions, and items using a 0 to 10 Likert scale to assess participants’ attitudes. The complete questionnaire instrument is available in Supplementary Material.

### Data analysis

A descriptive analysis was performed to summarize the current knowledge and perception of ADHD and autism in the workplace. Descriptive statistical techniques, including means and standard deviations (SD) for quantitative variables and absolute and relative frequencies for qualitative variables were used.

### Ethical considerations

This study complies with the principles of Spanish Law 14/2007 on Biomedical Research, which does not require Ethical Committee approval for studies involving anonymized surveys where participants cannot be identified. However, the Ethical Committee of the Hospital Universitario Infanta Leonor and Hospital Virgen de la Torre, issued a favorable report (Code 085 − 24), supporting the study’s compliance with regulatory standards. All personal data remained confidential, with survey responses anonymous. Participants were encouraged to answer honestly, and participation was voluntary and uncompensated. An electronic informed consent form was presented on the initial page of the survey, and all participants provided their consent before proceeding.

## Results

### Participant demographics

As shown in Table 1 and 880 participants completed the questionnaire, yielding a response rate of 75.3%. The sample included 62.1% females. Most participants were aged 30 to 50 years, with a mean age of 43 (SD = 9.6). The majority of respondents held advanced academic degrees (64.1%). Regarding nationality, most respondents were from Spain (87.6%, *n* = 764), with smaller representations from Brazil, the United Kingdom, and Italy, each accounting for less than 2% of the sample. Additionally, 61.9% of participants reported having children.

**Table 1:** Demographic characteristics of survey participants.

Category	*n* = 880
Language of questionnaire completion (*n* = 880), n (%) English (United States) Spanish (Spain)	82 (9.3%) 798 (90.7%)
Age (*n* = 876), mean (SD)	43 (9.6)
Gender (*n* = 878),n (%) Female Male Undeclared	545 (62.1%) 323 (36.8%) 10 (1.1%)
Country of origin (*n* = 872),n (%) Spain Brazil UK Italy Argentina Others	764 (87.6%) 13 (1.5%) 13 (1.5%) 12 (1.4%) 9 (1.0%) 61 (7.0%)
Highest level of education (*n* = 879),n (%) Postgraduate (Master’s degree or PhD) University degree Intermediate or higher degree Secondary education	563 (64.1%) 263 (29.9%) 43 (4.9%) 10 (1.1%)
Place of work (*n* = 878),n (%) Alexion Spain Alexion Global AstraZeneca Spain AstraZeneca Global Hub (Barcelona)	38 (4.3%) 38 (4.3%) 617 (70.3%) 185 (21.1%)
Having children (*n* = 879),n (%)	544 (61.9%)
Number of children (*n* = 539),mean (SD)	1.9 (0.7%)

SD: standard deviation; UK: United Kingdom.

### Knowledge of ADHD and ASD

**Fig. 1:**
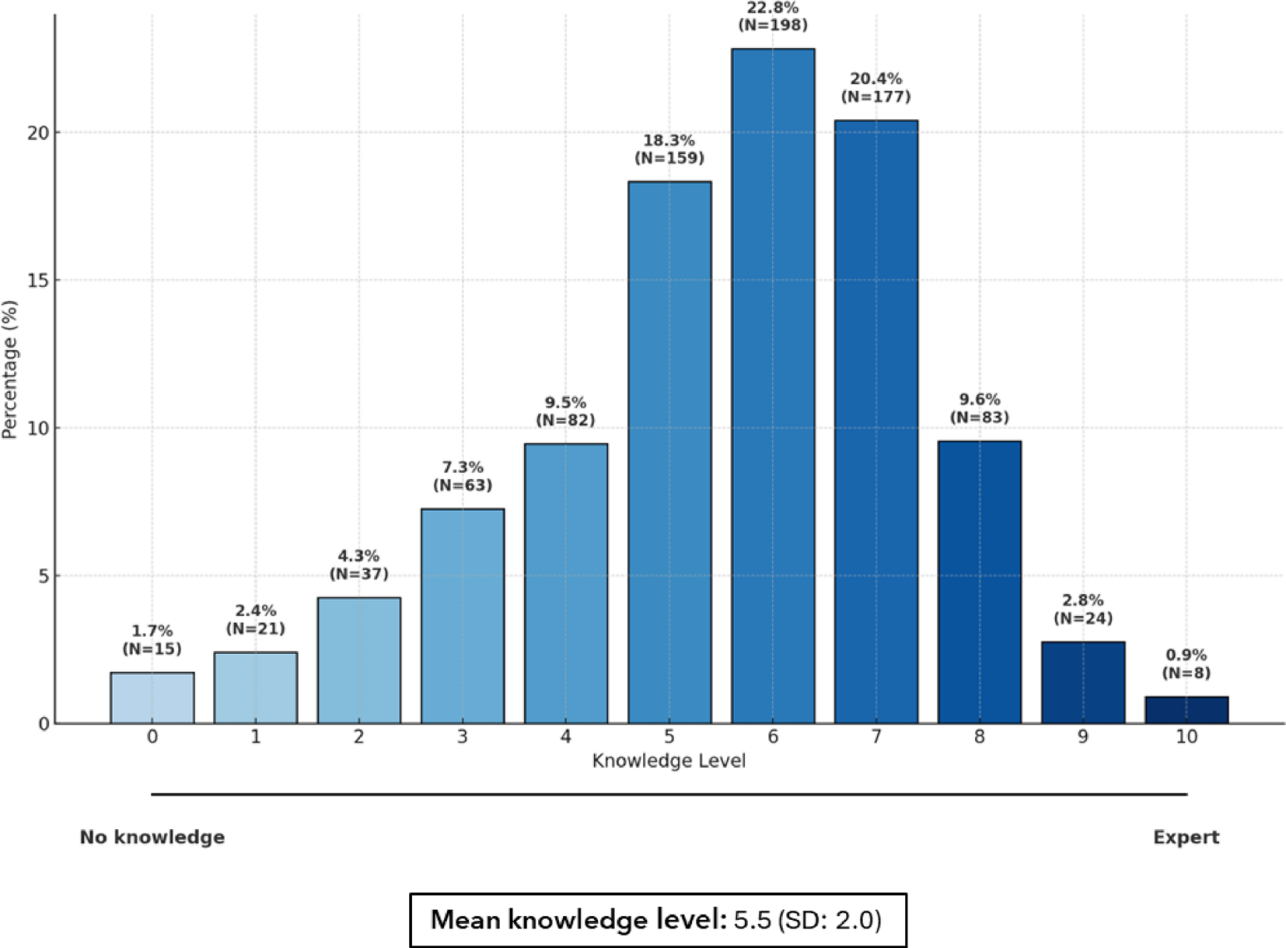
Knowledge levels on neurodevelopmental disorders (*N* = 867). SD: standard deviation.

The survey revealed substantial variability in employees’ knowledge of ADHD and autism, with a mean self-assessed knowledge score of 5.5 (SD = 2.0) on a scale from 0 to 10 (Fig. 1). Awareness of ADHD was high (98.9%), as was awareness of autism (98.1%). However, fewer respondents recognized intellectual developmental disorders (52.4%) or language disorders (66.7%) as part of the neurodevelopmental spectrum (Table 2**)**.

**Table 2:** Awareness of neurodevelopmental disorders among respondents.

Disorder	*N*	Percentage (%)
Autism	863	98.1
ADHD	870	99.0
Intellectual Development Disorder	461	52.4
Dyslexia	860	97.7
ASD	741	84.2

ADHD:Attention-Deficit/Hyperactivity Disorder; ASD:Autism Spectrum Disorder. “Autism” and “ASD” were included as separate response options to assess whether participants recognized both terms. Given common usage differences, some respondents may not realize they refer to the same condition.

**Fig. 2:**
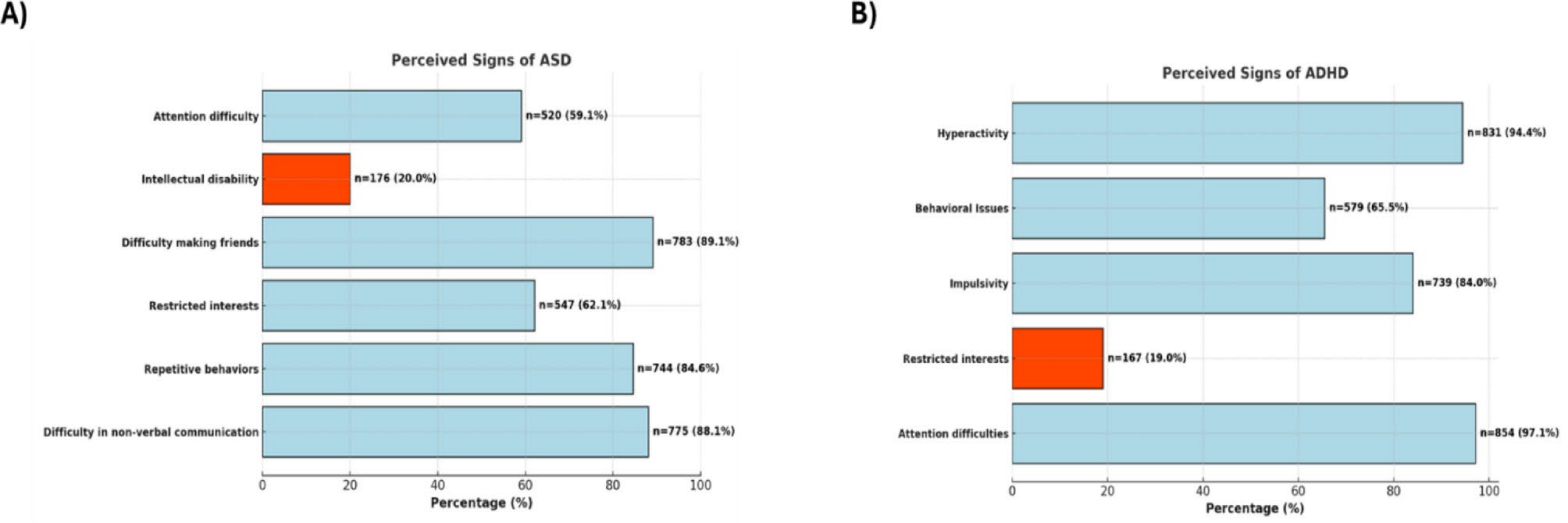
Perceived signs of autism (**A**) and ADHD (**B**). Misconceptions of the participants are highlighted in orange.

Most participants correctly identified common symptoms associated with ASD, including difficulties in making friends (89.0%), non-verbal communication challenges (88.1%), and repetitive behaviors (84.5%) (Fig. 2A). However, 20% incorrectly associated intellectual disability with autism. Similarly, respondents demonstrated knowledge of key ADHD symptoms, such as difficulty maintaining attention, hyperactivity, and impulsivity, but 19.0% incorrectly identified restricted interests as a sign of this disorder (Fig. 2B).

### Perceptions of neurodivergent individuals in the workplace

**Fig. 3:**
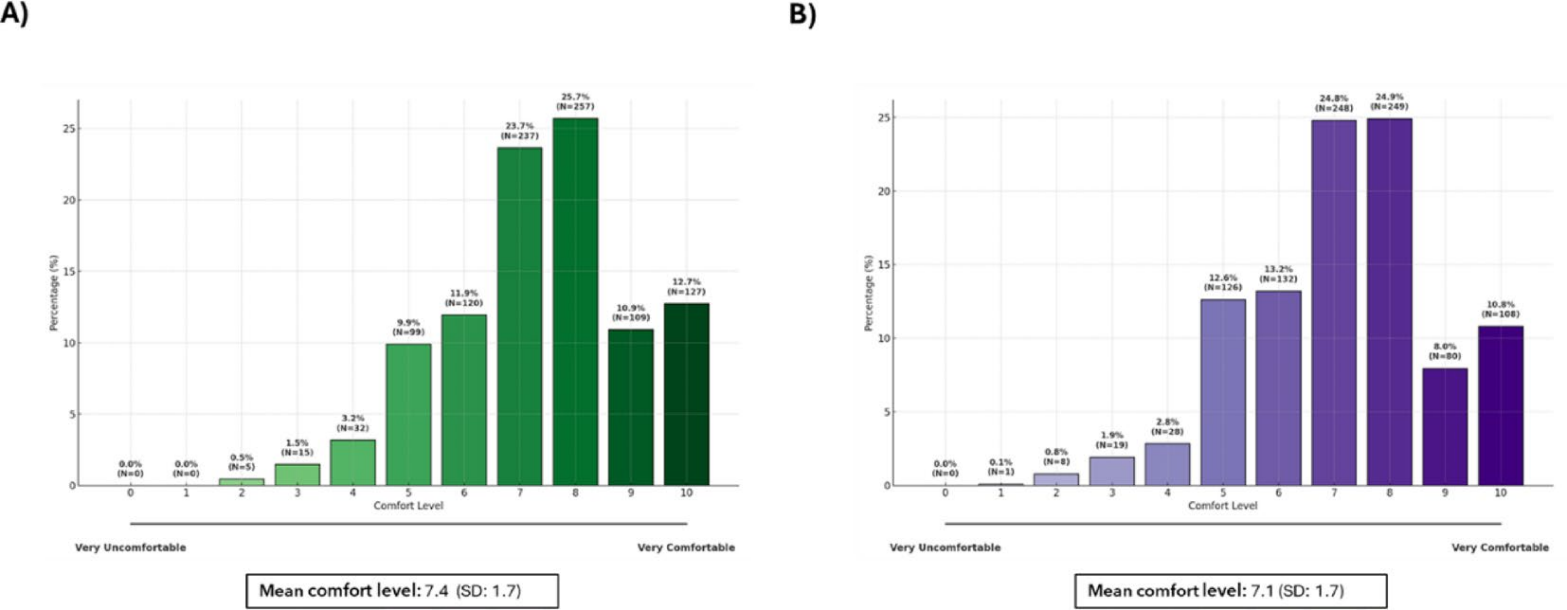
Comfort level for potentially working with a person with ADHD (**A**) or autism (**B**) (*N* = 879). SD: standard deviation.

Participants generally expressed positive attitudes toward working with neurodivergent individuals as shown in Fig. 3. On a scale of 0 (very uncomfortable) to 10 (very comfortable), the mean comfort level for potentially working with a person with autism was 7.1 (SD = 1.7), and 7.4 (1.7) for ADHD. Outside the workplace, comfort levels were similar, with a mean score of 7.6 (SD = 1.7) for autism and 7.9 (SD = 1.6) for ADHD.

**Fig. 4:**
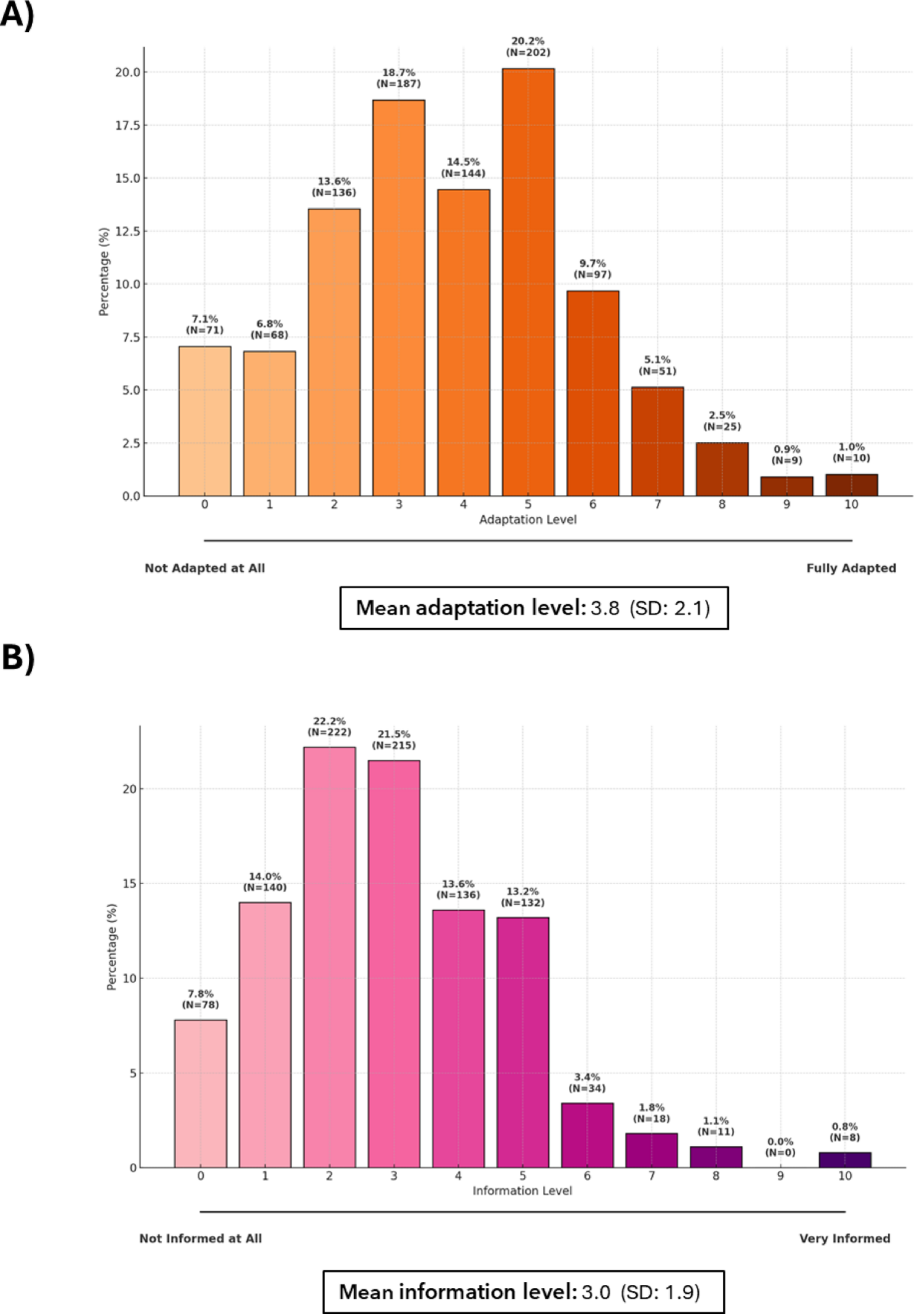
Perceptions of workplace adaptation (A: *N* = 878) and society awareness (B: *N* = 879) regarding neurodivergent individuals. SD: standard deviation.

Despite these positive attitudes, 60.6% of participants believed that workplaces were insufficiently adapted to accommodate neurodivergent individuals (≤ 4 on the scale), with a mean rating of 3.8 (SD = 2.1) on a scale of 0 to 10 (Fig. 4A). Additionally, 79.2% felt that society, in general, is not well-informed about neurodevelopmental disorders, with a mean societal knowledge score of 3.0 (1.9) out of 10 (Fig. 4B).

### Sources of information and familiarity with neurodevelopmental disorders

**Fig. 5:**
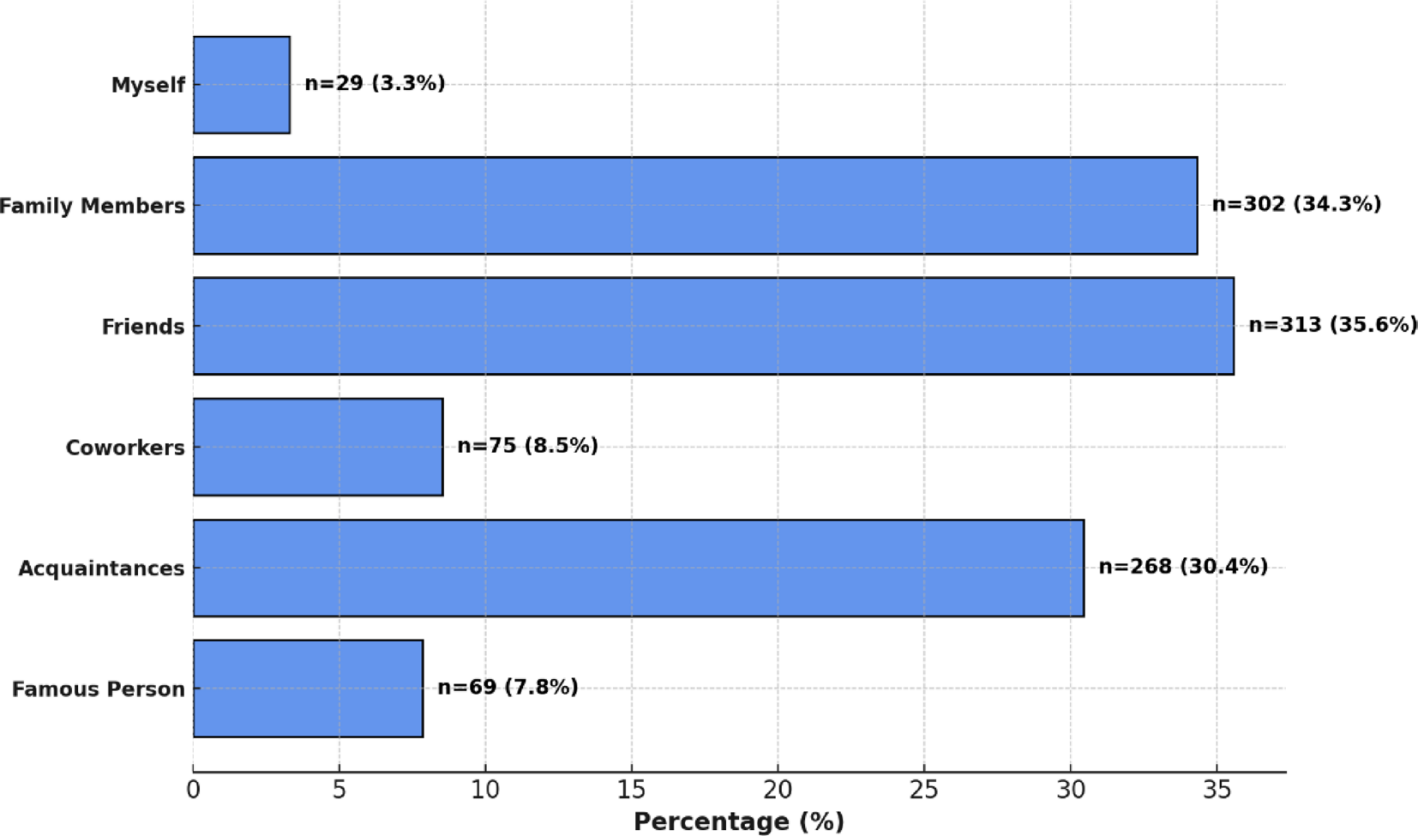
Reported connections to individuals with neurodevelopmental disorders.

Participants were asked to identify the sources from which they had learned about neurodevelopmental disorders, specifically ADHD and autism. The most frequently reported sources of information included friends or family members (76.8%), followed by the internet (62.6%), healthcare professionals (50.2%), and mass media (49.0%). Participants indicated a preference for educational initiatives, such as school talks (87.5%), social media (67.6%), workshops/seminars (57.6%), and patient associations (47.8%) (Table 3). When asked if they personally knew someone with a neurodevelopmental disorder, 72.9% responded affirmatively, with the most common connections being friends (35.6%) and family members (34.3%), while a smaller proportion reported knowing a coworker with such a disorder (8.5%) (Fig. 5).

**Table 3:** Sources and effective methods of information on neurodevelopmental disorders.

Source of information		
	*N*	Percentage %
Internet	551	62.6
Social media	339	38.5
Books	322	36.6
Friends or family	676	76.8
Healthcare professionals	442	50.2
Educational campaigns	255	29.0
Mass media (TV, radio, newspapers)	432	49.1
Patient associations	98	11.1

Effective means of information		
Specialized websites	413	46.9
Social media	595	67.6
Workshops and seminars	507	57.6
Press	401	45.6
School talks	770	87.5
Television	490	55.7
Patient associations	421	47.8

## Discussion

This study aimed to assess employee knowledge and perceptions of neurodivergent conditions, particularly ADHD and autism, in a multinational corporate context. The findings reveal significant gaps in the understanding of neurodiversity among well-educated respondents. Notably, persistent misconceptions about neurodevelopmental conditions remain prevalent, particularly regarding the diverse abilities of people with autism or ADHD. These findings underline the urgent need for targeted, impactful interventions.

The survey results show that while there is a general willingness to support neurodivergent colleagues, many respondents lack a deep understanding of these conditions. For instance, one in five respondents mistakenly identified restricted interests as a sign of ADHD. That is in agreement with previous studies showing that major misbeliefs about neurodevelopmental conditions still exist in the general population^22^. Our results align with those of Lord et al. (2020), who found that many people do not fully grasp the cognitive diversity among individuals with autism^23^.

The differences in perspectives highlighted by this study demonstrate a critical need for educational initiatives aimed at creating a genuinely inclusive workplace for neurodivergent employees. Our findings highlight a clear preference among employees for formats such as school talks, social media, workshops, and patient association events. These preferred formats should serve as a blueprint for designing future corporate training programs that prioritize awareness campaigns addressing widely held misconceptions and highlighting the strengths that neurodivergent individuals might bring to the Table^13^. In addition to awareness and education, adjustments in workplace policies, such as providing sensory-sensitive areas and accommodating flexible schedules, can substantially improve the work experience for neurodivergent individuals. Moreover, establishing support networks like peer mentoring programs could offer guidance and promote a sense of belonging within the organization, as mentioned in other studies.

Clear and effective communication could be another paramount factor for successful integration^24^. Guidelines on how to interact with colleagues who have ADHD or autism can equip employees with the skills to navigate various communication styles, such as interpreting non-verbal cues and supporting individuals with impulsivity-related difficulties^25,26^. Establishing a continuous feedback system may further encourage this inclusivity by allowing for monitoring and making adjustments^25,26^. We acknowledge that interventions focused solely on individual disclosure may be insufficient^27,28^. Therefore, integrating a universal design approach, such as implementing flexible policies and clear communication structures, could benefit all employees and reduce reliance on self-identification for access to support^28^. By cultivating a more adaptable environment, companies like AstraZeneca and Alexion can better support diversity in their workforce, aligning with broader corporate responsibility goals and enhancing overall team dynamics and organizational success. This is in line with the National Strategic Plan on Mental Health recommendations that advocate for the integration of health promotion^29^. The Plan specifically emphasizes the need for coordinated efforts to include community-based support systems and workplaces to reduce stigma and improve mental health outcomes^29^.

Recent discussion in this area is the apparent underestimation of the unique strengths that neurodivergent individuals often bring to the workplace. Incorporating neurodiverse talent not only promotes equity but also presents a valuable opportunity for companies. Neurodivergent individuals often excel in areas such as meticulous attention to detail, inventive thinking, and practical problem-solving, which can drive innovation and significantly enhance team performance^14^. Examples from companies like SAP and Microsoft show that simple adjustments, such as providing noise-canceling headphones, can help neurodivergent individuals thrive^11,30^. Cultivating a culture that celebrates and develops neurodivergent talents enables organizations to harness this often-overlooked talent pool. However, the main challenge lies in reframing the narrative. ADHD and autism are frequently seen through a lens of deficit, focusing more on individuals’ difficulties than their capabilities. Shifting this perspective can enable companies to recognize neurodivergent employees as critical agents to their success in the long term.

While our study provides significant insights, it also presents several limitations. Our sample was drawn from a single multinational pharmaceutical company and comprised participants with a high level of education, but this homogeneity may somewhat limit the generalizability of the findings. Nevertheless, these findings offer a glimpse into the realities faced even within some of the most favorable settings, i.e., an educated workforce in a progressive corporate environment. If misconceptions persist here, they are likely more profound in less privileged settings. Additionally, we did not collect data on the neurotype of participants, which means that some respondents may have been neurodivergent themselves. This limits our ability to interpret whether the perspectives captured reflect solely neurotypical views. Also, although the study was conducted in Spain, the participating company is part of a larger British/Swedish organization with a global presence and includes a subsidiary headquartered in the United States. As such, the corporate culture, internal communication policies, and diversity initiatives influenced by international leadership may have impacted participants’ perceptions of neurodiversity. This cultural and organizational influence might represent an important limitation when interpreting the results. Furthermore, although several open-ended items were included in the survey, we did not conduct qualitative analyses such as thematic analysis. This limits the depth of insight into how employees articulate their attitudes toward neurodivergence. Moreover, although we refer to substantial variability in employee knowledge, our analysis was strictly descriptive and did not include inferential statistical testing. A more detailed quantitative analysis is planned for a future manuscript. On the other hand, although previous surveys in the US and UK have explored neurodiversity in the workplace^31–34^, most have primarily emphasized the employer’s perspective and only gathered employees’ opinions on implementing neurodiversity programs, while failing to examine employees’ knowledge and opinions in greater depth. Therefore, to our knowledge, this study represents the first comprehensive survey specifically targeting employee perceptions of neurodiversity in the workplace, laying a solid groundwork for future studies.

Our study marks only the beginning of this journey. Future research should focus on the impact of educational interventions designed to improve the understanding and perceptions of neurodiversity in the workplace. Longitudinal studies must be conducted to track and monitor these transformations over time. Expanding this research to other sectors and countries—especially those with cultural or industry-specific challenges not represented here—should also be a priority. Our findings underscore the need for further research to better understand the experiences of neurodivergent individuals and to establish actionable recommendations.

In conclusion, this study highlights the ongoing challenges of neurodiversity in the workplace, particularly ADHD and ASD. While a willingness to support neurodivergent colleagues exists, considerable knowledge gaps remain. By proactively educating their personnel, creating adaptable settings, and encouraging transparent communication, companies can promote neurodivergent employees and release their full potential. As global personnel continue to diversify, businesses that embrace neurodiversity will be well-positioned to lead in innovation, creativity, and overall organizational success.

## Supplementary Information

Below is the link to the electronic supplementary material.

Supplementary Material 1

## Data Availability

The datasets used and/or analysed during the current study are available from the corresponding author on reasonable request.
