# Supplementary material for: A survey of knowledge and perceptions of ADHD and autism spectrum disorder in the workplace at a large corporation
Source: Sci Rep. 2025 Oct 2;15:34424. doi: 10.1038/s41598-025-17470-8 (PMC12491399; doi:10.1038/s41598-025-17470-8)
Supplement: Supplementary file 1 — Supplementary Material 1 [file 41598_2025_17470_MOESM1_ESM.docx]

**Supplementary Material**

1. Complete questionnaire

**Knowledge and awareness in the workplace about neurodevelopmental disorders: Attention Deficit Hyperactivity Disorder (ADHD) and Autism Spectrum Disorder (ASD)**

**A. Consent to data publication**

**1.** Are you in agreement with the data you provide in this survey being published in a scientific journal in an aggregated and anonymized manner? *

Yes

No

**B. Sociodemographic profile**

**2.** Personal identifier creation (it must be an 8-digit number):

a) The first 3 digits of your personal mobile number.
b) The month of your birth, with two digits, from 01 to 12 (e.g., 01 for January, 02 for February, etc.).
c) The last 3 digits of your ID number/passport. *

**3.** Age (in completed years)

**4.** Gender

- Female
- Male
- Prefer not to say
- Other

**5.** Country of origin

**6.** What is the highest level of education you have completed?

- Primary education
- Secondary education
- Medium or higher degree (vocational training, etc.)
- University degree (diploma, bachelor’s degree)
- Postgraduate studies (master’s or doctorate)

**7.** Where do you work?

- AstraZeneca Spain
- AstraZeneca Global Hub (Barcelona)
- Alexion Spain
- Alexion Global

**8.** Do you have children?

- Yes
- No

**9.** How many? (Enter a number equal to or greater than 1)

**C. General knowledge**

**10.** What is your level of knowledge about neurodevelopmental disorders?
(0 to 10 scale, from 'I know nothing' to 'I recognize myself as an expert')

**11.** Have you heard of the following disorders? (Check all that apply)

- Autism
- Attention Deficit Hyperactivity Disorder (ADHD)
- Intellectual Development Disorder
- Dyslexia
- Autism Spectrum Disorder (ASD)
- Language Disorder

**12.** Have you heard of other neurodevelopmental disorders?

- Yes
- No

**13.** Which ones?

**D. Specific Knowledge**

**14.** What do you think are some of the signs/symptoms of autism? (Check all that apply)

- Difficulty in nonverbal communication
- Repetitive behaviors
- Restricted interests
- Difficulty making friends
- Intellectual disability

**15.** What do you think are some of the main symptoms/signs of ADHD? (Check all that apply)

- Attention difficulties
- Restricted interests
- Impulsivity
- Behavioral disorder
- Hyperactivity

**E. Awareness and Attitudes**

These questions explore attitudes and perceptions towards individuals with neurodevelopmental disorders.

**16.** How comfortable would you feel working with a person with autism?
(0 to 10 scale, from 'Very uncomfortable' to 'Very comfortable')

**17.** How comfortable would you feel working with a person with ADHD?
(0 to 10 scale, from 'Very uncomfortable' to 'Very comfortable')

**18.** How comfortable would you feel interacting with a person with autism outside of work?
(0 to 10 scale, from 'Very uncomfortable' to 'Very comfortable')

**19.** How comfortable would you feel interacting with a person with ADHD outside of work?
(0 to 10 scale, from 'Very uncomfortable' to 'Very comfortable')

**20.** Do you think companies/workplaces are sufficiently adapted for neurodevelopmental disorders?
(0 to 10 scale, from 'Not adapted at all' to 'Very adapted')

**21.** Do you think society is sufficiently informed about neurodevelopmental disorders?
(0 to 10 scale, from 'Not informed at all' to 'Very informed')

**F. Sources of Information**

**22.** Where have you obtained information about neurodevelopmental disorders? (Check all that apply)

- Internet
- Social media
- Books
- Friends or family
- Health professionals
- Educational campaigns
- Media (TV, radio, newspapers)
- Patient associations

**23.** Which media do you consider most effective for educating the population about neurodevelopmental disorders? (Check all that apply)

- Specialized websites
- Social media
- Workshops and seminars
- Press
- School talks
- Television
- Patient associations

**G. Other Comments**

**24.** Do you know anyone in your environment who has a neurodevelopmental disorder?

- Yes
- No

**25.** Who do you know in your environment with a neurodevelopmental disorder? (Check all that apply)

- Myself
- Family members
- Friends
- Coworkers
- Acquaintances
- A famous person

**26.** What suggestions do you have to improve awareness of neurodevelopmental disorders in the workplace?
